# Neuropathologic damage induced by radiofrequency ablation at different temperatures

**DOI:** 10.1016/j.clinsp.2022.100033

**Published:** 2022-04-15

**Authors:** Yu Dong, Ying Chen, Baoguo Yao, Peng Song, Ruiting Xu, Rui Li, Ping Liu, Yu Zhang, Li Mu, Xin Tong, Linwei Ma, Jianjun Yu, Li Su

**Affiliations:** Department of Thyroid Breast, People's Hospital of Ningxia Hui Autonomous Region, Yinchuan, China

**Keywords:** Neuropathologic damage, Temperatures, Animal model

## Abstract

**Objective:**

To explore the molecular mechanism of neuropathologic damage induced by radiofrequency ablation at different temperatures.

**Methods:**

This is basic research, and 36 SD rats were used to construct the neuropathological injury model. The rats were subjected to radiofrequency stimulation at different temperatures and were divided into 6 groups according to the temperature injury: 42°, 47°, 52°, 57°, 62°, and 67°C groups. Conduction time, conduction distance, and nerve conduction velocity were recorded after temperature injury. HE-staining was used to observe the histopathological morphology of the sciatic nerve. The expression of SCN9A, SCN3B, and NFASC protein in sciatic nerve tissue were detected by western blot.

**Results:**

With the increase in temperature, nerve conduction velocity gradually decreased, and neurons were damaged when the temperature was 67°C. HE-staining showed that the degrees of degeneration of neurons in rats at 47°, 52°, 57°, 62°, and 67°C were gradually increased. The expression of SCN9A, SCN3B protein in 57°, 62°, 67°C groups were much higher than that of NC, 42°, 47°, 52°C groups. However, the expression of NFASC protein in 57°, 62°, 67°C groups was much lower than that of the NC, 42°, 47°, 52°C groups.

**Conclusion:**

There was a positive correlation between temperature caused by the radiofrequency stimulation to neuropathological damage. The mechanism is closely related to the expression of SCN9A, SCN3B, and NFASC protein in nerve tissue caused by heat transfer injury.

## Highlights

• There is a positive correlation between temperature and neuropathological damage caused by RFA at different temperatures.
• There is a positive correlation between nerve conduction velocity and temperature.
• Nerve injury occurs when the temperature reaches 67°C, and the main pathogenesis is closely related to the expression of SCN9A, SCN3B and NFASC protein in sciatic nerve tissue caused by heat transfer injury.

## Introduction

Radiofrequency Ablation (RFA) is a minimally invasive technique that has been developed in recent years. It is also a form of thermal ablation. The main principle is that the ions in the tissues constantly vibrate under the action of the electricity of the radiofrequency electrode, resulting in friction and increasing the temperature around the tissues.^1^ RFA applies radio frequency electricity to the human body due to the body is an electrical conductor. When the radio frequency electricity flows through the local tissue, organizations can make the local tissue temperature increase due to the thermal effect. After reaching a certain temperature, the water inside and outside the cell evaporates and dries. At the same time, the cell protein denatures to make its activity and function of cell lose and achieve the purpose of treatment.^2^ Radiofrequency refers to the electromagnetic wave whose frequency is in a certain range. At present, there is no clear dividing standard, and the frequency range of 200∼750 KHz is often used in medical applications.^3^^,^^4^ RFA now has been widely used in clinical therapy and achieved good efficacy in therapy of liver cancer, lung cancer, and other diseases.^5^, ^6^, ^7^ However, the mechanisms of neurological disease and neuropathologic injury have not been reported.

Transfer of heat to surrounding tissue during ablation of diseased tissue can lead to thermal damage of recurrent laryngeal nerve. In clinical practice, controlling the temperature to ensure complete ablation of the lesion without causing damage to the peripheral tissues and nerves is still a problem worth studying. At the same time, what is the temperature threshold of nerve tolerance and whether the temperature can be controlled within this threshold to avoid the occurrence of thermal nerve injury are worth studying. In this study, the sciatic nerve region of rats was surgically exposed to thermal burn to simulate clinical treatment of the lesion in this region. The degree of sciatic nerve injury caused by thermal burn was observed by observing the changes in nerve electrophysiology and histology. To clarify the ""heat transfer damage effect"" of radiofrequency ablation is important. Therefore, it is essential to guide clinicians to control the clinical radiofrequency ablation threshold.

## Materials and methods

### Materials

SD rats were half male and half female, a total number of 36, weighed 250 to 350 g, and were 9 to 10 weeks old. Animal Use License: SCXK (Ningxia) 2020-0001. The rats were provided by the Animal Experiment Center of Ningxia Medical University, and the experimental rats were uniformly fed chow provided by the animal Experiment Center. Feeding conditions: the temperature was (22°± 2°C), relative humidity was 40%‒50%, free feeding and drinking water. Scalpel cutting, vernier caliper, and HE-staining kit were purchased from Beyotime Biotechnology.

Rat anti-human primary antibody SCN9A, SCN3B, NFASC (1:500), HRP labeled sheep anti-rat secondary antibody (1:5, 000) (Beyotime Biotechnology); sterile pipette: 5 mL/10 mL (Costar, USA); liquid transfer gun: 10 μL/20 μL/200 μL/1000 μL (Eppendorf); centrifuge tube: 10 mL/50 mL (Thermo fisher); centrifuge with low temperature and high speed (Eppendorf, USA); confocal microscope (Olyplus, Japan); Leica optical microscope (Leica, German); western blot instruments (Eppendorf).

### Methods

#### Grouping

After one week of adaptive feeding, 36 SD rats were randomly stimulated with a self-made temperature injury probe for 10s, and the temperature was recorded respectively. According to the temperature damage, they were divided into 6 groups with 6 rats in each group: 42°, 47°, 52°, 57°, 62°, and 67°C group. At the same time, a normal control group was set without temperature damage intervention. All animal experiments shall be carried out in accordance with the national regulations on medical animal experiments and shall be subject to the supervision and inspection of the Ethics committee at any time.

#### Neuropathological injury model and conduction time

After abdominal anesthesia, the rats were placed on an animal experiment table. The rat skin was cut open, the sciatic nerve of the hind limb was exposed, and the piroid muscle was severed to expose the visual field of the central segment of the sciatic nerve. The nerve stimulation probe was placed at the efferent side of the rat sciatic nerve (sciatic notch). The signal was received at the sciatic nerve of the rat ipsilateral ankle joint with the receiving probe electrode. The reference electrode was placed between the nerve stimulation probe and the nerve receiving probe, 1 cm away from the receiving probe. The self-made temperature injury probe was connected with thermostatic hot water and placed 1 cm away from the nerve stimulation probe. BL-420E biological function experimental system software was used to control and adjust the stimulus intensity repeatedly. The stimulus intensity when the composite action potential appeared or disappeared was used as the stimulus threshold. The threshold multiple was used as the stimulus intensity, and the time value after temperature damage was recorded.

#### Conduction distance

The hind limbs of the rats were straight extended longitudinally along the passage and direction of the sciatic nerve. The distance between the stimulus probe and the receiving probe was measured using a vernier caliper.

#### Nerve conduction velocity

Nerve Conduction Velocity (NCV) was calculated as the following equation: NCV (cm/ms) = conduction distance/conduction time.

### HE-staining

The rats were sacrificed by excessive anesthesia, and the sciatic nerve tissue damaged by labeled heat was removed. The rats were fixed in the supine position on the operating table, and a 1 cm incision was made along the direction of the sciatic nerve at the junction of the left gluteus and femoral. The piriformis muscle was obtusely separated and exposed with the hemostatic forceps. The sciatic nerve was free with the glass minute hand, and the sciatic nerve was intercepted 5 mm above and below the injury for reserve. Then, the skin and muscles in the middle of the rat's back were cut longitudinally and stripped to expose the spine. The spinal cord was located at L4‒L5 and removed. Sciatic nerve tissue was washed with normal saline at 4°C and fixed with neutral formalin for 12h. Then the tissue was dehydrated by gradient ethanol, further dipped in wax, embedded, sliced, with a thickness of 4∼5 μm. Staining was performed according to the procedure of the HE-staining kit, and the histopathological morphology of the sciatic nerve was observed under a microscope.

#### Western blot

Total proteins of tissue were extracted from heat damage to the sciatic nerve tissue of each group of rats, and 20 μg proteins were sampled. Concentrated gel (5%) and isolated gel (12%) were prepared respectively to isolate proteins by SDS-PAGE. Objective and internal proteins were transferred to NC membrane, then closed with 5% skimmed milk powder sealing fluid for 2h at room temperature. Rat anti-Human primary antibody Rat anti-Human primary antibody SCN9A, SCN3B, NFASC (1:500), and Rat anti-Human primary antibody β-actin (1:1000) were added and incubated at 4°C overnight. TBST was washed 4 times, then HRP labeled Sheep anti Rat secondary antibody (1:5, 000) was added and incubated at 37°C for 1h. TBST was washed 4 times. The color was developed with ECL luminescent solution, protein bands were exposed by gel image analysis system, and images were photographed and quantitatively analyzed. The experiment was repeated three times.

#### Statistical analysis

All experiments were repeated independently at least three times. One-way Analysis of Variance (ANOVA) was used to analyze the data, which was expressed as the mean ± Standard Deviation (SDD). Statistical significance was defined as p < 0.05.

## Results

### Conduction time

The conduction time value after temperature damage was recorded in Table 1. The conduction time of NC, 42°, and 47°C groups were close, and the values were 0.27 ± 0.12, 0.32 ± 0.14, and 0.37 ± 0.11 ms, respectively. Among them, the value of the NC group was the smallest, but there was no statistical difference between the NC, 42° and 47°C group. However, the conduction time of the 52°C group was 0.48 ± 0.15 ms, which was much lower than that of the 57°C group (0.96 ± 0.23 ms) and 62°C group (1.7 ± 0.20 ms). There were obvious differences in the 52°, 57°, and 62°C groups compared with the NC group. Moreover, the value of the group at 67°C could not be recorded, indicating that the neuron had been damaged.

**Table 1:** Conduction time in different temperature groups.

Group	NC	42°C	47°C	52°C	57°C	62°C	67°C
Time (ms)	0.27±0.12	0.32±0.14	0.37±0.11	0.48±0.15^a^	0.96±0.23^b^	1.7±0.20^b^	Unable to record

Note: ^a^ p < 0.01, ^b^ p < 0.001, compared with NC group.

### Conduction distance

The vernier caliper was used to measure the conduction distance between the stimulus probe and the receiving probe, as shown in Table 2. The conduction distance of NC, 42°, 47°, 52°, and 57°C groups were 2.73 ± 0.36, 5.64 ± 0.50, 2.69 ± 0.80, 2.70 ± 0.68, 2.8 ± 0.52 cm, respectively. However, when the temperature reached 62°C, the distance was shortened to 1.5 ± 0.34 cm, which was significantly lower than the previous groups, and exhibited a statistical difference. Moreover, when the temperature was 67°C, the distance between the stimulus probe and the receiving probe could not be measured, indicating that the neuron had been damaged.

**Table 2:** Conduction distance at different temperature groups.

Group	NC	42°C	47°C	52°C	57°C	62°C	67°C
Distance (cm)	2.73±0.36	2.64±0.50	2.69±0.80	2.70±0.68	2.8±0.52	1.5±0.34^a^	Unable to record

Note: ^a^ p < 0.01 compared with NC group.

### Nerve conduction velocity

**Fig. 1:**
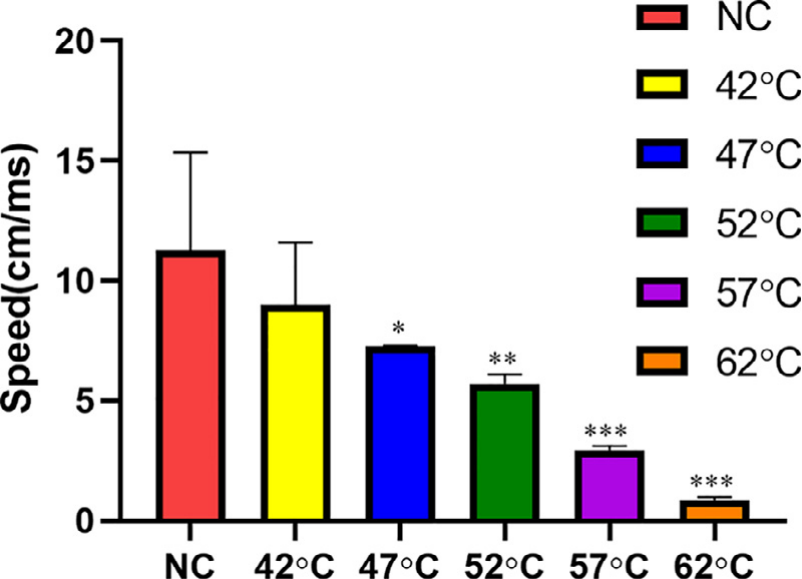
Nerve conduction velocity in different temperature groups (n = 3). *^⁎⁎^*p < 0.01, *^⁎⁎⁎^*p < 0.001 compared with NC group.

Detailed results of nerve conduction velocity are shown in Fig. 1. In the NC, 42°, 47°C group, the nerve conduction velocity was 11.28 ± 4.07, 8.99 ± 2.61, 7.27 ± 0.01 cm/ms, respectively. There was no significant statistical difference between the NC and 42°C groups. When the temperature was 52°C, the nerve conduction velocity (5.71 ± 0.38 cm/ms) was significantly lower than that at 42° and 47°C, and the difference was statistically significant compared with NC group. When the temperature reached 67°C, the speed could not be calculated because the time and distance could not be recorded. The above indicators indicate neuronal necrosis at 67°C.

### HE-staining

**Fig. 2:**
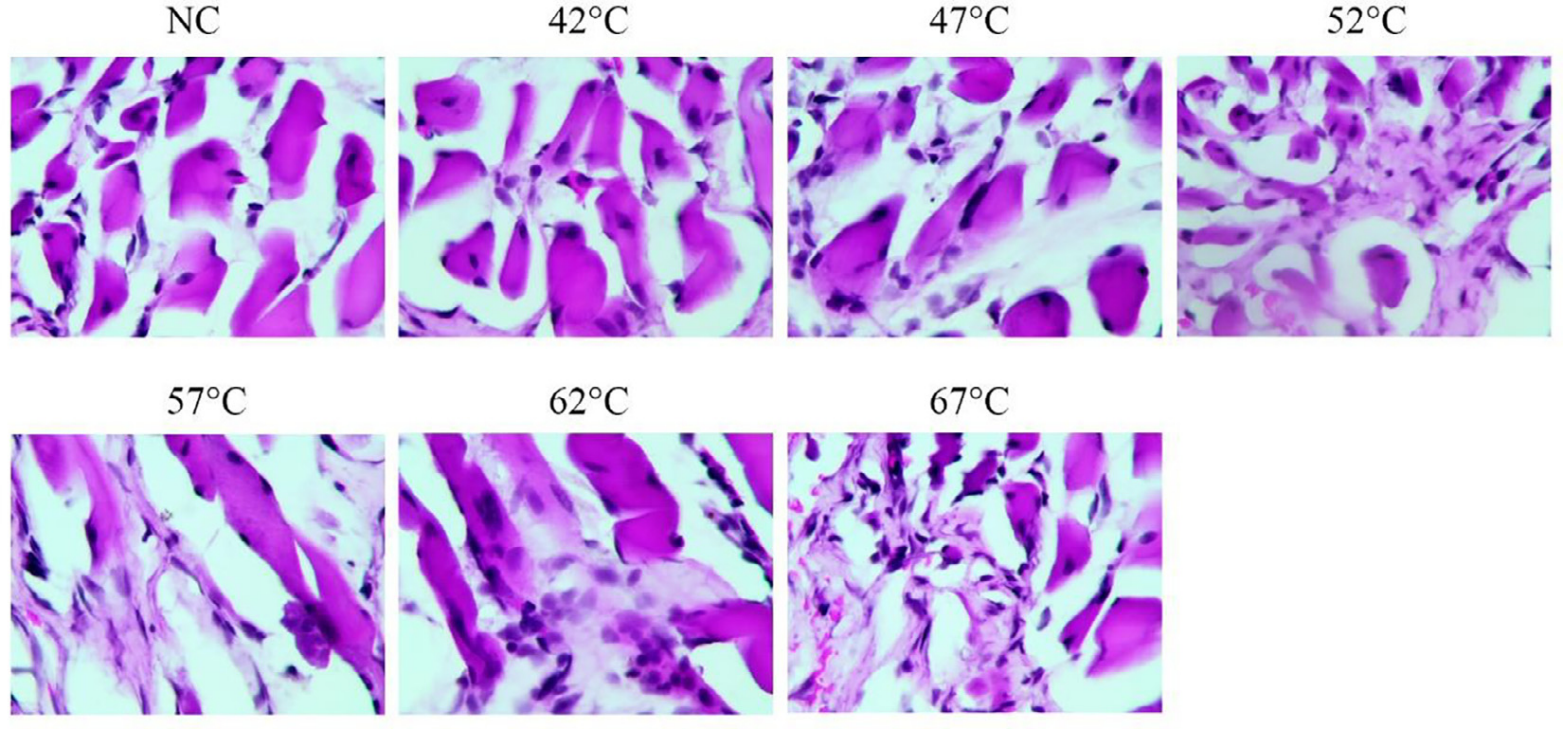
HE-staining of sciatic nerve tissue in different groups (400 ×).

In the normal group and the 42°C group, the nuclei of rat sciatic neurons were centered, and nishite was massive. However, in the 47°, 52°, 57°, 62°, 67°C groups, the degree of degeneration of rat neurons was gradually aggravated, the number of neurons decreased, intracellular vacuoles increased, and the cytoplasm shrank. In addition, definite coagulation necrosis occurred at 67°C, and the cell structure was destroyed, especially the nuclear changes (Fig. 2).

### Western blot

**Fig. 3:**
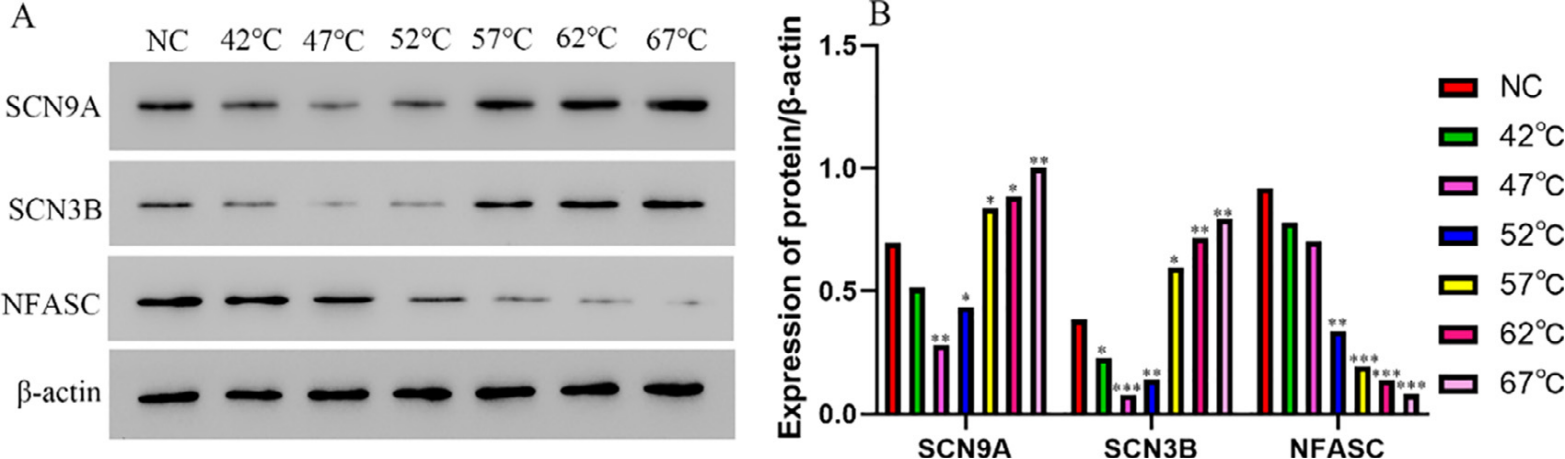
Protein level of SCN9A, SCN3B, NFASC in different groups determined by Western blotting (Mean ± SD, n = 3). A, Gray value; B, Statistical analysis. *^⁎⁎^*p < 0.01, *^⁎⁎^*p < 0.01, *^⁎⁎⁎^*p < 0.001 compared with NC group.

The expression of SCN9A, SCN3B, NFASC in Sciatic nerve tissue in different groups was shown in Fig. 3. The expression of SCN9A, SCN3B protein in 57°, 62°, 67°C groups were much higher than that of NC, 42°, 47°, 52°C groups (p < 0.05). The expression of SCN9A, SCN3B protein in 57°, 62°, 67°C groups has a positive correlation with temperature. The higher the temperature is, the more expression is. However, the expression of NFASC protein in 57°, 62°, 67°C groups was much lower than that of NC, 42°, 47°, 52 °C groups (p < 0.01).

## Discussion

Radiofrequency Ablation (RFA) is considered to be a kind of thermal damage caused by thermal energy generated by electric current passing through tissues.^8^ RFA is characterized by excessive local temperature, causing damage to the lipid bilayer structure of cells, cell membrane rupture, and cell lysis.^9^ A temperature injury probe was placed at the efferent site (sciatic notch) of the rat sciatic nerve. The signal was received at the sciatic nerve of the rat ipsilateral ankle joint with the nerve receiving probe electrode. The present research results showed that the conduction time of the NC group was similar to the 42° and 47°C groups, and there was no statistical difference between the three groups. The conduction time of the 52°C group was 0.48±0.15 ms, which was significantly lower than that of the 57°C (0.96±0.23 ms) and 62°C (1.7±0.20 ms) group. The value of the group at 67°C could not be recorded, indicating that the neuron had been damaged. The conduction distance and nerve conduction velocity had the same trend with conduction time.

Nerve tissue has the lowest resistance in human tissue, so the electric current through it is the strongest, and the tissue is most vulnerable to damage. The pathological manifestations of peripheral nerve after electric burn were cell disintegration, axonal degeneration, nerve fiber swelling, and demyelination.^10^ HE-staining showed that in the 47°, 52°, 57°, 62°, and 67°C groups, the degree of degeneration of rat neurons was gradually aggravated, the number of neurons decreased, intracellular vacuoles increased, and the cytoplasm shrank. In addition, definite coagulation necrosis occurred at 67°C, and the cell structure was destroyed, especially the nuclear changes.

The changes in cell function and the damage to the cell membrane after RFA have been widely observed, but the changes in macromolecular functional proteins on the membrane and their effects on cell function have not been studied. There are various kinds of neural ion channels. SCN9A ion channel, as a large molecular protein, is bound to cause changes in its structure and function when it is damaged by electricity. In addition, with the increase of temperature-induced by voltage and electricity, nerve damage is gradually strengthened.^11^

Studies have shown that the Sodium Channel B3 subunit (SCN3B) is up-regulated in the dorsal root ganglion during nerve damage.^12^ A series of plasticity changes occur in each subunit of SCN3B ion channel in the pathogenesis of neuropathic pain.^13^ If SCN3B expression is decreased throughout the body, other complications may appear. It has been found that SCN3B knockout mice show changes in the electrophysiological characteristics of the heart.^14^ Animal studies have shown that SCN3B is up-regulated after the preparation of multiple neuropathic pain models.^12^ Western blot indicated that the expression of SCN9A, SCN3B protein in 57°, 62°, 67°C groups were much higher than that of NC, 42°, 47°, 52°C groups. The higher the temperature was, the higher the expression of SCN3B protein was, which also indicated neuropathy damage. Therefore, the authors hypothesize if the expression of SCN9A and SCN3B protein is downregulated, it could inhibit the neuropathy response, such as nerve injury and nerve pain. This can be a potential target for treating a neuropathic injury.

Neurofascin (NFASC) is a transmembrane protein and also an important glial cell adhesion molecule, which expresses primarily at peripheral and central myelin axon-like junctions.^15^ NFASC is abundant in the adult central nervous system, especially in the cerebellum and peripheral nerves.^16^^,^^17^ NFASC mutations have recently been associated with neurodevelopmental disorders. NFASC plays a key role in the development and function of the AIS and Ranvier nodes.^18^^,^^19^ The main role of NFASC is to connect the extracellular matrix and the intracellular skeleton of glial cells and neurons.^20^, ^21^, ^22^ In adult animals, chronic intrathecal injection of antibodies also leads to NFASC loss and changes in motor nerve conduction.^23^ Western blot has detected the expression of NFASC protein in the sciatic nerve tissue damaged by RFA. It has been found that the expression of NFASC protein in 52°, 57°, 62°, 67°C group were much lower than that of NC, 42°, 47°C group. The loss of NFASC expression caused a barrier to the formation of the initial axons, resulting in significant ataxia and decreased nerve conduction rate in rats. Moreover, NFASC also plays an important role in the formation of myelin sheath structures and post-injury repair.^24^

## Conclusion

There is a positive correlation between temperature and neuropathological damage caused by RFA at different temperatures, but there is a positive correlation between nerve conduction velocity and temperature. Nerve injury occurs when the temperature reaches 67°C, and the main pathogenesis is closely related to the expression of SCN9A, SCN3B, and NFASC protein in sciatic nerve tissue caused by heat transfer injury.

## Ethics approval and consent to participate

The ethical approval was obtained from the Ethics Committee of People's Hospital of Ningxia Hui Autonomous Region.

## Consent to publish

All of the authors have Consented to publish this research.

## Availability of data and materials

The data are free to access and available upon request.

## Authors' contributions

YD, YC, BGY: Conceived and designed experiments; YD, YC, BGY: Performed experiments and data analysis; PS, RTX, RL, PL, YZ, LM, XT, LWM, JJY, LS: Provided technical support, data collection and analysis; and YD, YC, BGY: Wrote the manuscript. All authors provided final approval for the submitted and published version.

## Declaration of Competing Interest

The authors declare no conflicts of interest.
